# Flavonoid-Rich Extract of *Actinidia macrosperma* (A Wild Kiwifruit) Inhibits Angiotensin-Converting Enzyme In Vitro

**DOI:** 10.3390/foods7090146

**Published:** 2018-09-05

**Authors:** Sujeewa K. Hettihewa, Yacine Hemar, H. P. Vasantha Rupasinghe

**Affiliations:** 1 School of Chemical Sciences, University of Auckland, 23 Symonds Street, Auckland 1142, New Zealand; krishanthi2001@yahoo.com (S.K.H.); y.hemar@auckland.ac.nz (Y.H.); 2 Department of Plant, Food, and Environmental Sciences, Faculty of Agriculture, Dalhousie University, Truro, NS B2N 5E3, Canada

**Keywords:** ACE, *Actinidia macrosperma*, flavonoids, polyphenols, hypertension, kiwifruit

## Abstract

Increasing interest in flavonoids in kiwifruit is due to the health-promoting properties of these bioactives. Inhibition of the angiotensin-converting enzyme (ACE) is one of the main therapeutic targets in controlling hypertension. The present study investigated the ACE inhibitory activity of flavonoid-rich extracts obtained from different kiwifruit genotypes. The flavonoid-rich extracts were prepared from fruits of *Actinidia macrosperma*, *Actinidia deliciosa* cv Hayward (Green kiwifruit), and *Actinidia chinensis* cv Hort 16A (Gold kiwifruit) by steeping the lyophilized fruit samples in 70% aqueous acetone, followed by partitioning the crude extracts with hexane. The composition of each extract was analyzed using ultrahigh-performance liquid chromatography-mass spectrometry (UPLC-MS/MS). The ACE inhibitory activity of the fruit extracts was performed using a fluorescence-based biochemical assay. The subclass flavonol was the most abundant group of flavonoids detected in all the extracts tested from three different kiwifruit cultivars. Quercetin-3-*O*-galactoside, quercetin-3-*O*-glucoside, quercetin-3-*O*-rhamnoside, quercetin-3-*O*-rutinoside, quercetin-3-*O*-arabinoglucoside, catechin, epigallocatechin gallate, epigallocatechin, chlorogenic, ferulic, isoferulic, and caffeic acid were prominent phenolics found in *A. macrosperma* kiwifruit. Overall, the flavonoid-rich extract from *A. macrosperma* showed a significantly (*p* < 0.05) high percentage of inhibition (IC_50_ = 0.49 mg/mL), and enzyme kinetic studies suggested that it inhibits ACE activity in vitro. The kiwifruit extracts tested were found to be moderately effective as ACE inhibitors in vitro when compared to the other plant extracts reported in the literature. Further studies should be carried out to identify the active compounds from *A. macrosperma* and to validate the findings using experimental animal models of hypertension.

## 1. Introduction

Hypertension has become a common risk factor for cardiovascular disease around the world, which affects all ages, from children to adults [1]. It is reported that the angiotensin-converting enzyme (ACE) plays an important role in the renin–angiotensin aldosterone system (RAAS) by cleaving angiotensin I to angiotensin II, which is responsible for increasing blood pressure [2,3]. Thus, inhibition of ACE has been identified as a major therapeutic target for controlling over-activation of RAAS. Prescription drugs such as captopril, ramipril, lisinopril, and enalaprilare are synthetic ACE inhibitors, which have been widely used in the treatment of hypertension [4]. Because these drugs are often reported to have undesirable side effects, interest in searching natural sources of ACE inhibitors has increased. Most studies have shown that plant extracts, which are rich in specific peptides and flavonoids are found to be effective as natural ACE inhibitors [5,6,7,8,9,10,11,12,13,14,15,16]. Flavonoid-rich fruits and vegetables and their products have now gained attention for their capability to manage blood pressure [17,18,19,20].

Kiwifruit has a reputation for being particularly nutritious and medicinally important [21,22,23,24,25]. The most commonly consumed kiwifruits in the world are Green (*Actinidia deliciosa* (A. Chev.) ‘Hayward’) and Zespri^®^ Gold kiwifruit (*A. sinensis* Planch. ‘Hort16A’) [21]. A few other varieties (e.g., baby kiwifruit) are grown commercially, and a number of other varieties are currently being assessed for future commercialization. *Actinidia macrosperma* is a noncommercial type of kiwifruit which is orange and small-sized with large seeds, and has relatively thick, highly-colored, hairless skin. It is reported that the roots and stems of these plants have been extensively employed to treat various ailments, such as leprosy, abscess, rheumatism, arthritis inflammation, jaundice, and abnormal leucorrhoea, in Chinese traditional medicine [26].

The present study investigated the in vitro ACE inhibitory activity of flavonoid-rich extracts obtained from kiwifruit cultivars, namely *A. macrosperma*, *A. deliciosa* (Hayward), and *A. chinensis* (Hort 16A), using a fluorescence-based biochemical assay, followed by determination of the kinetic parameters of the inhibition.

## 2. Methodology

### 2.1. Chemicals

The ACE extracted from rabbit lung, histidine-l–hippuryl-l–leucine–chloride (HHL), histidine leucine (His–Leu), NaOH, HCl, ethanol anhydrous, captopril, *O*-phaldialdehyde, dimethyl sulfoxide, and HPLC grade methanol were purchased from Sigma–Aldrich Canada Ltd., Oakville, ON, Canada. Borate saline buffer (100 mM boric acid, 1.5 M sodium chloride, sterile, pH adjusted to 8.3) was purchased from Teknova, Hollister, CA, USA. All other reagents and consumables were purchased from Fisher Scientific, Ottawa, ON, Canada.

### 2.2. Plant Materials

The fruits of *A. macrosperma* were collected between April and August 2010 at the research orchard of Plant and Food Ltd. in Te Puke Bay, New Zealand. *A. deliciosa* and *A. chinensis* were purchased at a market in Auckland, New Zealand.

### 2.3. Preparation of Flavonoid-Rich Kiwifruit Extracts

The lyophilized ground fruit sample from *A. macrosperma* kiwifruit (5 g) was steeped in 70% aqueous acetone (100 mL) in a Scott Duran bottle (250 mL) for 6 hours in the dark, with nitrogen gas purging at 30 °C. The extract was filtered through a glass filter and the filtrate was collected in an ice bath. The residue was subjected to re-extraction, and then filtrates were combined together and concentrated on a rotary evaporator (Buchi, New Zealand) below 35 °C under a vacuum. The crude extracts from *A. deliciosa* and *A. chinensis* kiwifruits were also prepared using the same extraction procedure described above. The flavonoid-rich extracts were obtained by partitioning the crude extracts with hexane. The composition of each extract was analyzed using ultra-high performance liquid chromatography-mass spectrometry (UPLC-MS/MS).

### 2.4. UPLC-MS/MS Analysis of Phenolics in the Kiwifruit Extracts

Analyses of major individual phenolic compounds present in the flavonoid-rich kiwifruit extracts were performed at the Department of Plant, Food, and Environmental Sciences, Faculty of Agriculture, Dalhousie University, Truro, Nova Scotia, Canada, according to the procedure reported by Rupasinghe et al. [27]. All analyses were performed using a Waters H-class UPLC separations module (Waters, Milford, MA, USA), coupled with a Quattro *micro* API MS/MS system and controlled with Masslynx V4.0 data analysis system (Micromass, Cary, NC, USA). The column used was an Aquity BEH C_18_ (100 mm × 2.1 mm × 1.7 µm) (Waters, Milford, MA, USA). For the separation of the flavonol, flavan-3-ol, phenolic acid, and dihydrochalcone compounds, a gradient elution was carried out, with 0.1% formic acid in water (solvent A) and 0.1% formic acid in acetonitrile (solvent B) at a flow rate of 0.3 mL/min. A linear gradient profile was used, with the following proportions of solvent A applied at time *t* (min) (*t*, A%): (0, 94%), (2, 83.5%), (2.61, 83%), (2.17, 82.5%), (3.63, 82.5%), (4.08, 81.5%), (4.76, 80%), (6.75, 20%), (8.75, 94%), (12, 94%).

Electrospray ionization in negative ion mode (ESI-) was used for the analysis of the flavonol, flavan-3-ol, phenolic acid, and dihydrochalcone compounds. The following conditions were used: Capillary voltage −3000 V, and nebulizer gas (N_2_) temperature 375 °C at a flow rate of 0.3 mL/min. The cone voltage (25 to 50 V) was optimized for each compound. Multiple reactions–monitoring (MRM) mode using specific precursor/product ion transitions was employed for quantification in comparison with standards: *m*/*z* 301→105 for quercetin (Q), *m*/*z* 609→301 for Q-3-*O*-rutinoside, *m*/*z* 463→301 for Q-3-*O*-glucoside and Q-3-*O*-galactoside, *m*/*z* 448→301 for Q-3-*O*-rhamnotoside, *m*/*z* 595→301 for Q-3-*O*-peltoside, *m*/*z* 273→167 for phloritin, *m*/*z* 435→273 for phloridzin, *m*/*z* 353→191 for chlorogenic acid, *m*/*z* 179→135 for caffeic acid, *m*/*z* 193→134 for ferulic acid and isoferulic acid, *m*/*z* 289→109 for catechin and epicatechin, and *m*/*z* 305→125 for epigallocatechin. The quantification of each analyte was performed using calibration curves created using the external standards.

### 2.5. Assay for ACE Inhibitory Activity

The in vitro ACE inhibitory activity of flavonoid-rich extracts prepared was performed according to the methods published by Cinq-Mars et al. [9] and Balasuriya and Rupasinghe [15], with slight modifications. The ACE enzyme inhibition assay was carried out with the presence of 2.5 mU ACE in buffer (30 µL), 0.78 mM HHL in buffer (150 µL), sodium borate buffer (pH 8.3) (9 µL), and different concentrations of test compounds (21 µL) in the Eppendorf tubes. All the experimental units, including testing units, negative control (without ACE and inhibitors), positive control (with ACE but no inhibitors), and the standard solution made of 10 mg/L captopril in 10% DMSO in buffer (with ACE), were run in triplicates for each experiment. All the experimental units were incubated at 37 °C using a shaker oven (Model: HP 50, Apollo Instrumentation for Molecular Biology, San Diego, CA, USA) for 1 h, followed by adding 0.35 M NaOH (150 µL) to stop the enzyme activity in the experimental unit. The formation of His–Leu by the cleavage of HHL in the presence of ACE was quantified through a spectrophotometric method based on fluorescence (excitation at 360 nm and emission at 500 nm). The mean fluorescence values of the samples were obtained in triplicates, and the percentage of inhibition of the enzyme was expressed in comparison with the positive control (Equation (1)):

Percent enzyme inhibition (%) = (1 − (F_sample_ − F_sample blank_)/F_positive control_) × 100 (1)

where F = fluorescence.

Dose-responsive enzyme inhibition was determined using different concentrations of each extract. The concentration of the tested extracts which could inhibit 50% of enzyme activity (IC_50_) was calculated using linear regression analysis plot of % ACE inhibition vs. concentrations of tested extract.

### 2.6. Determination of Kinetic Parameters of ACE Inhibition

Enzyme kinetic analysis for ACE activity was performed by following the method published by Balasuriya and Rupasinghe [15]. Briefly, each experimental unit consisted of 2.5 mU ACE in buffer (30 µL), the relevant concentration (0.125, 0.25, 0.5, 1, 2, 4, 8 mM) of HHL in buffer (150 µL), and sodium borate buffer (pH 8.3) (30 µL) in an Eppendorf tube. All the experimental units were incubated at 37 °C using a shaker oven (Model: HP 50, Appolo Instrumentation for Molecular Biology, CA, USA) for 1 h, followed by adding 0.35 M NaOH (150 µL) to stop the enzyme activity in the experimental unit. A known concentration of extracts obtained in 70% aqueous acetone from *A. macrosperma*, *A. deliciosa*, and *A. chinensis* kiwifruit was subjected to enzyme kinetic analysis for the ACE activity of inhibitors. Kinetic parameters were calculated by adjusting curves to the Michaelis–Menten kinetic equation (Equation (2)):

V_0_ = V_max_ (S)/(*K*_m_ + (S)) (2)

where V_0_ is the initial reaction rate, V_max_ is the maximum reaction rate, *K*_m_ is the Michaelis–Menten constant, and S is substrate concentration. The reaction rate of formation of His–Leu was plotted against the different substrate concentrations to obtain the saturation curves to derive Lineweaver–Burk double reciprocal plots to determine the type of the inhibition (Equation (3)):

1/*V* = (*K*_m_/V_max_) (1/*S*) + 1/V_max_ (3)

*K*_i_ (dissociating constant) was determined using the following equation:

m_i_ = m (1 + (I)/*K*_i_) (4)

where m_i_: slope from linear plot from the inhibited reaction, m: slope from linear plot from the noninhibited reaction, [I]: concentration of inhibitor, *K*_i_: dissociating constant of the inhibitor (inhibitory constant).

### 2.7. Statistical Analysis

All measurements were conducted in triplicate, and the results were expressed as mean ± SD. The effect of kiwifruit cultivar on the percentage of inhibition of ACE was analyzed through analysis of variance (ANOVA), using Originpro8 software (Origin Lab, Northampton, MA, USA). Pair wise multiple comparisons were evaluated based on Tukey’s significance difference test used in origin. Differences at *p* < 0.05 were considered significant.

## 3. Results and Discussion

### 3.1. ACE Inhibition

**Figure 1:**
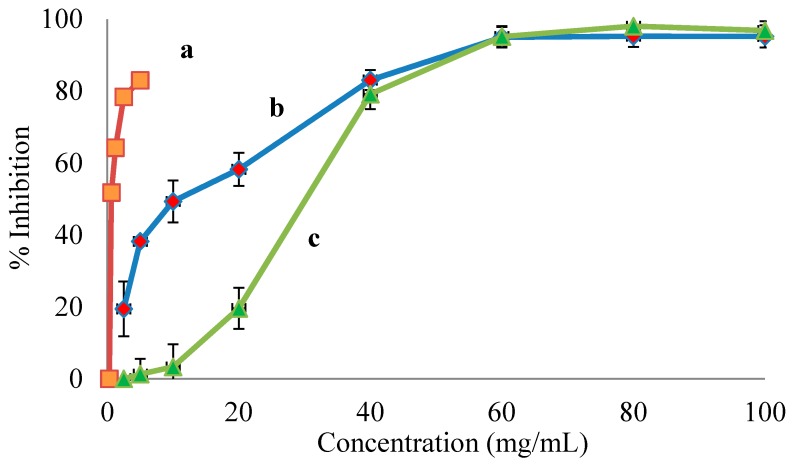
The dose–response curves of the percentage of angiotensin-converting enzyme (ACE) activity inhibition by the extracts of different kiwifruit genotypes: (**a**) *A. macrosperma* (wild kiwifruit); (**b**) *A. chinensis* cv Hort 16A (gold kiwifruit); and (**c**) *A. deliciosa* cv Hayward (green kiwifruit).

Kiwifruits, namely *A. deliciosa* (Hayward), *A. chinensis* (Hort 16A), and *A. macrosperma*, have been evaluated for their many pharmacological applications towards the management and treatment of human diseases, including antimicrobial activity [28], antioxidant activity [21,22,23,29] immune modulatory activity [30], and anticancer activity [24]. As far as we are aware, there are no reports on ACE inhibitory activity found in kiwifruits. Therefore, the potential anti-hypertensive activity of fruit extracts from different kiwifruit genotypes grown in New Zealand was evaluated through the inhibition of ACE, a key regulatory enzyme of RAAS [3]. All tested extracts inhibited ACE activity in a dose-dependent manner (Figure 1), with different IC_50_ ranging from 0.49 to 69.54 mg/mL of flavonoid-rich extracts (Table 1). The potential anti-hypertensive activities of kiwifruit cultivars showed that *A. macrosperma* possesses the lowest IC_50_ compared to the other two commercially grown cultivars (Table 1). This, in theory, indicates that the flavonoid-rich extract obtained from *A. macrosperma* possesses strong anti-hypertensive agents. These observations were supported by the significantly higher total phenolic and total flavonoid contents determined by LC-MS/MS (Table 2). The subclass flavonol was the most abundant group of flavonoids detected in all extracts tested, which were obtained from three different kiwifruit cultivars. Quercetin, quercetin-3-galactoside, quercetin-3-glucoside, quercetin-3-rhamnoside, quercetin-3-rutinoside, quercetin-arabinoglucoside, catechin, epigallocatechin gallate, epigallocatechin, chlorogenic, ferulic, isoferulic, and caffeic acids were prominent phenolics found in *A. macrosperma* kiwifruit. Some of these phenolics might be responsible for the strong ACE inhibition activity determined in the extract of *A. macrosperma* fruit. This observation is well supported by the literature, indicating that quercetin, quercetin sugar derivatives, and flavan-3-ols such as catechins, epigallocatechin gallate, and epigallocatechin show ACE inhibitory activity [15,31].

However, there are various reports that demonstrated flavonoids and flavonoid-rich plant extracts inhibit ACE activity. Loizzo et al. [10] reported hypertensive in vitro activities of the MeOH extracts and some flavonoids, namely apigenin, luteolin, kaempferol-3-*O*-α-arabinopyranoside, kaempferol-3-*O*-β-galactopyranoside, and quercetin-3-*O*-α-arabinopyranoside, isolated from *Ailanthus excelsa* (Roxb). The ACE inhibitory properties of flavonoid-rich apple peel extracts and selected quercetin derivatives have been reported [15]. The research findings carried out by Persson et al. [32] showed a dose-dependent ACE inhibition of major flavan-3-ols, namely, catechins, (−)-epicatechins, (−)-epigallocatechin, (−)-epicatechin gallate, and (−)-epigallocatechin gallate, isolated from green and black tea using a human umbilical vein endothelial cells (HUVEC) culture model. It has been reported that aqueous extracts of *Ginkgo biloba*, which is rich in quercetin derivatives as the major flavonoids, has a higher ACE inhibitory activity than that of ethanol extracts [33]. Oh et al. reported that fractions of stonecrop (*Sedum sarmentosum*) and five purified flavonols had ACE inhibitory properties [5]. However, one of the limitations of in vitro assays of ACE inhibitory activity of flavonoids-rich extracts is that most of the flavonoids exist as their metabolites in the central circulation.

ACE is a zinc-containing peptidyldipeptide hydrolase where the active site is known to have three parts. Therefore, it is well reported that the ACE inhibitory in vitro activity of flavonoids may be due to the formation of chelate complexes with the zinc atom within the active center of zinc-dependent metallopeptidases, or possibly due to the formation of hydrogen bridges between the inhibitor and phenolics near at the active site [34]. Thus, the presence of phenolic and flavonoid content in the extract would have contributed towards ACE inhibition.

### 3.2. Determination of Kinetic Parameters of ACE Inhibition

To study the type of inhibition of the ACE activity, enzyme kinetic studies were performed. Table 3 shows the kinetics of ACE activity without an inhibitor and in the presence of a known concentration of *A. macrosperma* (2.64 mg/L), *A chinensiscv* Hort 16 A (13 mg/mL), and *A. deliciosa* cv Hayward (31 mg/mL). ACE activity showed a Michaelis–Menten mechanism. The kinetic parameters obtained from these curves are shown in Table 3. The maximum rates of substrate hydrolysis (V_max_) and Michaelis–Menten constant (*K*_m_) were determined to characterize the kind of inhibition exerted by the extracts. The V_max_ was not significantly altered by each inhibitor, which suggests that it was a noncompetitive inhibitor for ACE. These findings are in agreement with the studies on flavonoid-rich apple peel extracts [15], flavan-3-ols, and anthocyanins-rich food extracts [6,34].

## 4. Conclusions

Several biological activities have already been reported for kiwifruit cultivars, such as antioxidant, anticancer, anti-inflammatory, and antimicrobial activities. This is the first report on a study supporting the potential anti-hypertensive activities of kiwi fruits. The results of this study clearly indicate that the flavonoid-rich extract from *A. macrosperma* shows potential as a food or nutraceutical source of anti-hypertensive agents. Overall, the flavonoid-rich extract from *A. macrosperma* showed a significantly (*p* < 0.05) high percentage of inhibition (IC_50_ = 0.49 mg/mL) in vitro. Kinetic determinations suggested that the flavonoids-rich extract obtained from *A. macrosperma* kiwifruit possibly inhibits enzyme activity either through nonspecific binding to the enzyme or by competing with the substrate for the active side. The subclass flavonol was the most abundant group of flavonoids detected in the tested extracts of three different kiwifruit genotypes. Quercetin-3-galactoside, quercetin-3-glucoside, quercetin-3-rhamnoside, quercetin-3-rutinoside, quercetin-arabinoglucoside, catechin, epigallocatechin gallate, epigallocatechin, chlorogenic, ferulic, isoferulic, and caffeic acid were prominent phenolic compounds found in *A. macrosperma* kiwifruit. Further investigations using experimental animal models and human clinical trials are required to explore the anti-hypertensive properties of *A. macrosperma***.**

## Figures and Tables

**Table 1:** IC_50_ of ACE inhibitory activity of extracts from different kiwifruit genotypes.

Kiwifruit Genotypes	IC_50_ (mg/mL)
*A.* *macrosperma*	0.49
*A.chinesis* cv Hort 16A	12.81
*A.deliciosa* cv Hayward	30.49

**Table 2:** Polyphenols content of three kiwifruit genotypes measured using UPLC-MS/MS.

Group/Name of the Flavonoid	Concentration of Phenolics (µg/g DW)		
	*A. macrosperma*	*A. chinensis*	*A. deliciosa*
Flavonol			
Quercetin-3-*O*-Galactoside	470.9	205.19	441.39
Quercetin-3-*O*-Glucoside	4.16	0.45	0.14
Quercetin Arabinoglucoside	2.53	0.06	0.21
Quercetin-3-*O*-Rhamnoside	2.99	0.61	0.31
Quercetin	2.56	nd	0.17
Quercetin-3-*O*-Rutinoside	1.96	0.29	0.55
Flavanol			
Epigallocatechin	1.55	0.61	0.46
Catechin	54.31	0.75	0.30
Epicatechin	0.91	5.15	0.74
Epigallocatechingallate	0.75	nd	0.40
Dihydrochalcones			
Phloridzin	3.12	2.03	5.08
Phloritin	0.14	0.21	0.14
Phenolic acids			
Chlorogenic acid	1.97	0.39	0.28
Caffeic acid	1.64	0.04	0.08
Ferulic acid	4.70	0.42	0.60
Isoferulic acid	32.71	15.12	28.17
Total Phenolics	586.9	231.32	479.02

DW: dry weight of the fruit; nd: not detected. UPLC-MS/MS: ultrahigh-performance liquid chromatography-mass spectrometry.

**Table 3:** Enzyme kinetic parameters of ACE inhibition by the extracts of three different kiwifruit genotypes.

Extract	Concentration Tested (mg/mL)	*K*_m_ (mM)	V_max_ (mM/min)	*K*_i_ (mg/mL)
No inhibitor	0	0.074	0.024	
*A. chinesis* cv Hort 16A	13.00	2.036	0.033	44.516
*A. deliciosa* cv Hayward	31.00	4.849	0.033	64.041
*A. macrosperma*	2.60	7.258	0.069	78.312
